# A pharmacological chaperone improves memory by reducing Aβ and tau neuropathology in a mouse model with plaques and tangles

**DOI:** 10.1186/s13024-019-0350-4

**Published:** 2020-01-22

**Authors:** Jian-Guo Li, Jin Chiu, Mercy Ramanjulu, Benjamin E. Blass, Domenico Praticò

**Affiliations:** 1 Alzheimer’s Center at Temple, Lewis Katz School of Medicine, Temple University, 3500 North Broad Street, MERB, suite 1160, Philadelphia, PA 19140 USA; 2 Moulder Center for Drug Discovery Research, School of Pharmacy, Temple University, Philadelphia, PA 19140 USA

**Keywords:** Alzheimer’s disease, Transgenic mice, Retromer complex, Amyloid beta, tau protein, Therapeutics

## Abstract

**Background:**

The vacuolar protein sorting 35 (VPS35) is a major component of the retromer complex system, an ubiquitous multiprotein assembly responsible for sorting and trafficking protein cargos out of the endosomes. VPS35 can regulate APP metabolism and Aβ formation, and its levels are reduced in Alzheimer’s disease (AD) brains. We and others demonstrated that VPS35 genetic manipulation modulates the phenotype of mouse models of AD. However, the translational value of this observation remains to be investigated.

**Methods:**

Triple transgenic mice were randomized to receive a pharmacological chaperone, which stabilizes the retromer complex, and the effect on their AD-like phenotype assessed.

**Results:**

Compared with controls, treated mice had a significant improvement in learning and memory, an elevation of VPS35 levels, and improved synaptic integrity. Additionally, the same animals had a significant decrease in Aβ levels and deposition, reduced tau phosphorylation and less astrocytes activation.

**Conclusions:**

Our study demonstrates that the enhancement of retromer function by pharmacological chaperones is a potentially novel and viable therapy against AD.

## Background

Alzheimer’s disease (AD) is a chronic neurodegenerative disorder characterized by memory impairments due to irreversible damages to neuronal cells [1]. Several studies have identified numerous genes as susceptibility risk factors for AD onset, including endosomal and vesicular trafficking genes such as the vacuolar protein sorting-associated 35 (VPS35) [2, 3]. VPS35 is the most important component of the cargo recognition core module of the retromer complex system, a ubiquitous multiprotein assembly whose main function is the trafficking of cargo proteins from the endosomes to the trans-Golgi or the cell plasma membrane [4]. Retromer complex dysfunction has been implicated in several neurodegenerative conditions such as Parkinson’s disease as well as AD. In particular, deficiency of VPS35 was reported in post-mortem AD brains, while in vitro studies showed that its down-regulation increases the Aβ formation [5, 6]. Additionally, genetic reduction of VPS35 in the Tg2576 mice, a model of AD-like brain amyloidosis, results in higher levels of Aβ peptides and amyloid plaques, cognitive impairments and synaptic dysfunction [7]. Interestingly, levels of VPS35 and the other two components of the retromer recognition core module (i.e., VPS26 and VPS29) are decreased in an age- and region-specific manner in the same mouse model, suggesting an early involvement of the system in the onset and development of the AD-like phenotype [8]. In our recent publication, we also demonstrated that a gain of function of VPS35 in the central nervous system of the triple transgenic mice rescued their phenotype including amelioration of long-term memory, lower Aβ levels, and decrease in pathological tau [9]. Taken together this evidence strongly supports a functional involvement of VPS35 and the retromer complex system in AD pathogenesis. However, whether this major component of the recognition core module is a potential target for pharmacological intervention in vivo remains to be investigated.

In the current paper we studied the effect of chronic administration of a pharmacological chaperone, TPT-172, on the development of the phenotype of a transgenic mouse model with memory impairments, Aβ deposits and tau tangles, the 3xTg mice. We selected this drug since previously it was reported to stabilize VPS35 against thermal denaturation, and by doing so to up-regulate its levels and restore the function of the entire complex system [10].

## Methods

### Animals

The 3xTg mice harboring a human mutant PS1 (M146 V) knock-in, and mutant amyloid precursor protein (APP; KM670/671NL) and tau (P301L) transgenes, and appropriate wild-type mice (WT) are the animals used in this study. A total of 34 three month-old mice were used for the study: ten WT (5 male and 5 female) and eight 3xTg (4 male and 4 female) mice were randomized to receive 75 mg/kg TPT-172 dissolved in their drinking water; whereas eight WT (4 male and 4 female) and eight 3xTg (4 male and 4 female) mice were randomized to receive drinking water and the vehicle in which the drug was dissolved (Ctrl). Fresh drinking water with newly dissolved drug or vehicle was prepared every other day. Throughout the paper the drug TPT-172 will be always referred as to TPT. Animals were treated for 9 months until they were 11–12 months old, when they first underwent behavioral testing, and then euthanized. During the study, the two groups of mice had access to food and water ad libitum, gained weight regularly, and did not manifest any apparent difference in terms of general health. All procedures were approved by the Institutional Animal Care and Usage Committee, in accordance with the U.S. National Institutes of Health guidelines.

### Behavioral tests

All animals were pre-handled for 3 days prior to testing. They were tested in a randomized order, and all tests were conducted by an experimenter blinded to the treatment or genotype.

#### Y-maze

The Y-maze apparatus consisted of 3 arms 32 cm long × 10 cm wide with 26 cm walls (San Diego Instruments, San Diego, CA). Testing was always performed in the same room and at the same time to ensure environmental consistency, as previously described [11, 12].

#### Fear-conditioning

The fear conditioning test paradigm was performed following methods previously described [11, 12]. Briefly, the test was conducted in a conditioning chamber (19 × 25 × 19 cm) equipped with black methacrylate walls, transparent front door, a speaker and grid floor (Start Fear System; Harvard Apparatus). On day one, mice were placed into the conditioning chamber and allowed free exploration for 2 min in the white noise (65 Db) before the delivery of the conditioned stimulus (CS) tone (30 s, 90 Db, 2000 Hz) paired with a foot-shock unconditioned stimulus (US; 2 s, 0.6 mA) through a grid floor at the end of the tone. A total of 3 pairs of CS-US pairing with a 30 s inter trial interval (ITI) were presented to each animal in the training stage. The mouse was removed from the chamber 1 min after the last foot-shock and placed back in its home cage. The contextual fear conditioning stage started 24 h after the training phase when the animal was put back inside the chamber for 5 min with white noise only (65 dB). The animal’s freezing responses to the environmental context were recorded. The tone fear conditioning stage started 2 h after the contextual stage. The animal was placed back to the same chamber with different contextual cues, including white wall, smooth metal floor, lemon extract drops, and red light. After 3 min of free exploration, the mouse was exposed to the exactly same 3 CS tones with 30 s ITI as in the training stage without the foot-shock and its freezing responses to the tones were recorded.

#### Morris water maze

To perform the Morris water maze we used a white circular plastic tank (122 cm in diameter, walls 76 cm high), filled with water maintained at 22° ± 2 °C, and made opaque by the addition of a nontoxic white paint, as previously described [11, 12]. Briefly, mice were trained for four consecutive days to find a Plexiglas platform submerged in water from four different starting points. Mice were then assessed in the probe trial, which consisted of a free swim lasting for 60 s without the platform, 24 h after the last training session. Animals’ performances were monitored using Any-Maze™ Video Tracking System (Stoelting Co., Wood Dale, IL).

### Immunoblot analyses

Primary antibodies used in this paper are summarized in Table 1. Proteins were extracted in enzyme immunoassay buffer containing 250 mM Tris base, 750 mM NaCl, 5% NP-40, 25 mM EDTA, 2.5% sodium deoxycholate, 0.5% sodium dodecyl sulfate, and an EDTA-free protease and phosphatase inhibitors cocktail tablet (Roche Applied Science, Indianapolis, IN), sonicated, and centrifuged at 45,000 rpm for 45 min at 4 °C, and supernatants were used for immunoblot analysis, as previously described [13–15]. Briefly, total protein concentration was determined by using a BCA Protein Assay Kit (Pierce, Rockford, IL), samples were electrophoretically separated according to the molecular weight of the target molecule, and then transferred onto nitrocellulose membranes (Bio-Rad). They were blocked with Odyssey blocking buffer for 1 h, and then incubated with primary antibodies overnight at 4 °C. After 3 washing cycles with T-TBS, membranes were incubated with IRDye 800CW-labeled secondary antibodies (LI-COR Bioscience, Lincoln, NE) at 22 °C for 1 h. Signals were developed with Odyssey Infrared Imaging Systems (LI-COR Bioscience). Actin was always used as an internal loading control.

**Table 1:** Antibodies used in the study

Antibody	Host	Application	Source	Catalog Number
4G8	Mouse	IHC	Covance	SIG-39220
ADAM10	Rabbit	WB	Millipore	AB19026
APH1	Rabbit	WB	Millipore	AB9214
APP	Mouse	WB	Millipore	MAB348
AT-180	Mouse	WB, IHC	Thermo	P10636
AT-270	Mouse	WB, IHC	Thermo	MN1050
AT-8	Mouse	WB, IHC	Thermo	MN1020
BACE-1	Rabbit	WB	IBL	18,711
cdk5	Rabbit	WB	Santa Cruz	sc-173
Cl-MPR	Rabbit	WB	AbCam	ab124767
CTFs	Mouse	WB	Millipore	MAB343
CTSD	Rabbit	WB	R&D system	AF1029
GFAP	Mouse	WB, IHC	Santa Cruz	sc-33,673
GSK 3α/β	Mouse	WB	Santa Cruz	sc-56,913
HT7	Mouse	WB, IHC	Thermo	MN1000
IBA1	Mouse	WB	Santa Cruz	sc-32,725
Nicastrin	Rabbit	WB	Cell Signaling	3632
p25/p35	Rabbit	WB	Santa Cruz	sc-820
Pen2	Rabbit	WB	Invitrogen	36–7100
pGSK 3α/β	Rabbit	WB	Cell Signaling	9331
PHF1	Mouse	WB, IHC	Dr. P. Davies	Gift
PHF13	Mouse	WB, IHC	Cell Signaling	9632
PP2A	Rabbit	WB	Thermo	PA5–17510
PS1	Rabbit	WB	Cell Signaling	3622S
PSD95	Mouse	WB	Thermo	MA1–045
s-APPα	Mouse	WB	IBL	11,088
s-APPβ	Mouse	WB	IBL	10,321
SorLA	Rabbit	WB	Cell Signaling	79,322
SYP	Mouse	WB, IHC	Santa Cruz	sc-12,737
VPS26	Goat	WB	Santa Cruz	sc-69,128
VPS29	Goat	WB	AbCam	ab10160
VPS35	Goat	WB, IF	AbCam	ab10099
β-Actin	Mouse	WB	Santa Cruz	sc-47,778

*WB* Western blot, *IHC* Immunohistochemistry, *IF* Immunofluorescence

### Biochemical analyses

Mouse brain homogenates were sequentially extracted first in radioimmunoprecipitation assay (RIPA) for the Aβ 1–40 and 1–42 soluble fractions, then in formic acid for the Aβ 1–40 and 1–42 insoluble fractions, and assayed by a sensitive sandwich enzyme-linked immunosorbent assay (ELISA) kit (Wako Chemicals, Richmond, VA) as previously described [13–15].

### Immunohistochemistry

Primary antibodies used are summarized in Table 1. Immunostaining was performed as reported previously by our group [13–15]. Briefly, serial coronal sections were mounted on 3-aminopropyl triethoxysilane-coated slides. Every eighth section from the habenular to the posterior commissure (8–10 sections per animal) was examined using unbiased stereological principles. The sections for testing Aβ (4G8 antibody) were deparaffinized, hydrated, and pretreated with formic acid (88%) and subsequently with 3% H_2_O_2_ in methanol. The sections for testing total tau (HT7 antibody), and phospho-tau epitopes, were deparaffinized, hydrated, subsequently pretreated with 3% H_2_O_2_ in methanol, and then treated with citrate (10 mM) or IHC-Tek Epitope Retrieval Solution (IHC World, Woodstock, MD) for antigen retrieval. Sections were blocked in 2% fetal bovine serum before incubation with primary antibody overnight at 4 °C. Next, sections were incubated with biotinylated anti-mouse immunoglobulin G (Vector Laboratories, Burlingame, CA) and then developed by using the avidin-biotin complex method (Vector Laboratories) with 3,3′-diaminobenzidine as a chromogen. Light microscopic images were used to calculate the area occupied by the immunoreactivities by using the software Image-Pro Plus for Windows version 5.0 (Media Cybernetics, Bethesda, MD).

### Immunofluorescent analysis

Immunofluorescence studies were performed as previously described [8]. Briefly, brain sections were deparaffinized, hydrated subsequently with 3% H_2_O_2_ in methanol, and then with citrate for antigen retrieval (10 mM). After 5 rinses with PBS, sections were incubated in a blocking solution (5% normal serum/0.4% TX-100) for 1 h at 22 °C and then with the primary antibody against VPS35 overnight at 4 °C. After washing with PBS, samples were incubated for 1 h with a secondary antibody donkey anti-goat IgG H&L (Alexa Fluor® 488). Coverslips were mounted using VECTASHIELD mounting medium (Vector Laboratories, Burlingame, CA, USA). Images were acquired using a NIKON Eclipse Ti2 with NIKON NIS-Elements AR 5.20.00 software, as previously described [8].

### Data analysis

Data were always collected and analyzed by an investigator who was blind about the treatment and or the genotype. One-way analysis of variance and then Bonferroni multiple comparison tests were performed using Prism 5.0 (GraphPad Software, La Jolla, CA). All data are always presented as mean ± standard error of the mean. Significance was set at *p* < 0.05.

## Results

### Administration of pharmacological chaperone ameliorates behavior

**Fig. 1:**
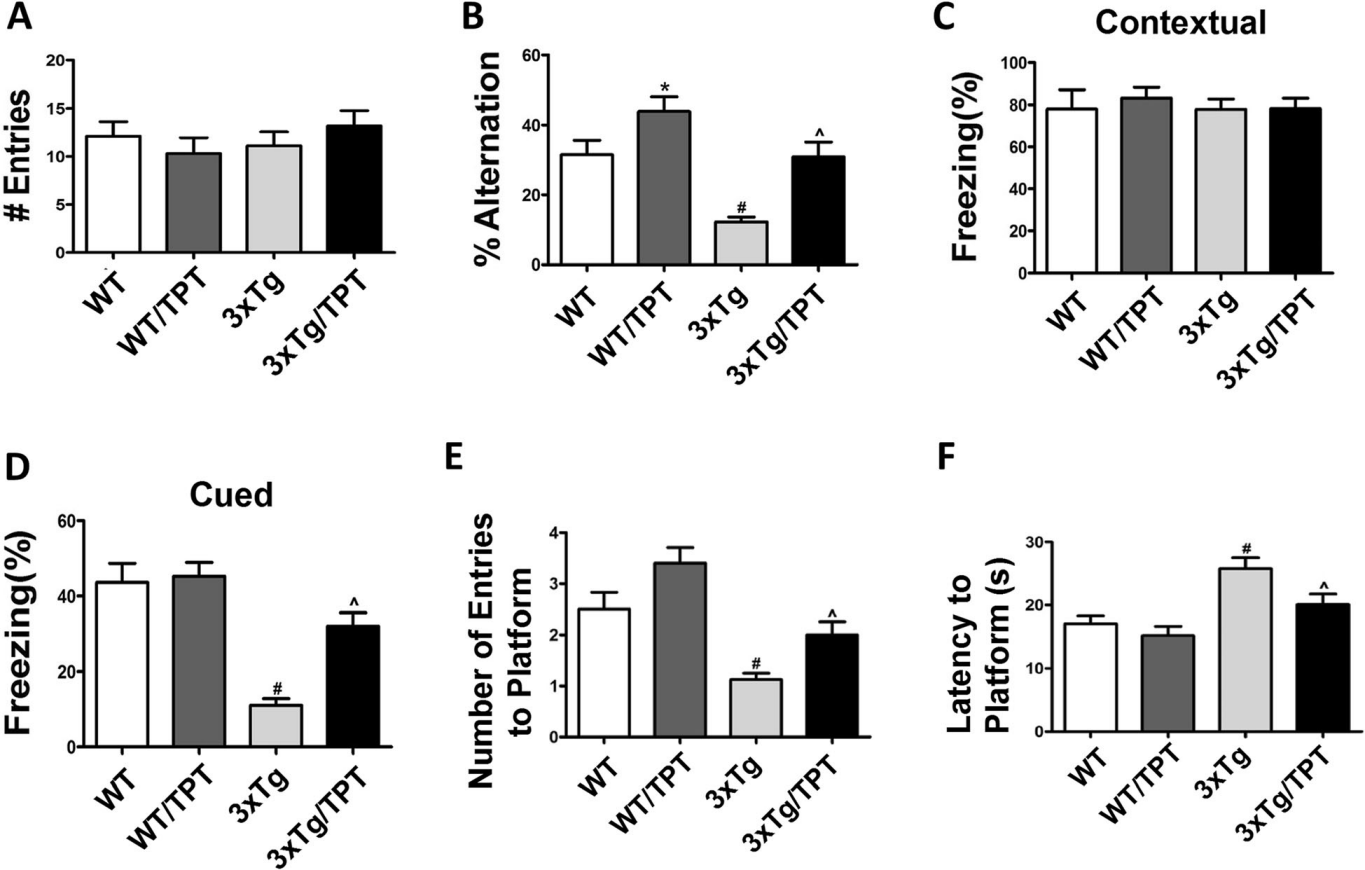
Pharmacological chaperone ameliorates behavioral deficits of 3xTg mice. **a** Number of total arm entries for wild-type mice (WT) and 3xTg mice (3xTg) treated with TPT or control (WT, 3xTg). **b** Percentage of alternations for WT and 3xTg mice treated with TPT or control. **c** Contexual fear memory response in WT and 3xTg mice treated with TPT or control. **d** Cued fear memory response in WT and 3xTg mice treated with TPT or control. **e** Morris water maze, probe trial for the same four groups of mice, number of entries to the platform area; **f** Morris water maze, probe trial for the same four groups of mice, latency to first entry to the platform area. Values represent mean ± standard error of the mean (*p < 0.05, WT Control vs WT/TPT; #*p* < 0.05, WT Control vs 3xTg Control; ^*p* < 0.05, 3xTg Control vs 3xTg/TPT). (WT Control: *n* = 10; WT/TPT: *n* = 8; 3xTg Control, n = 8; 3xTg/TPT, n = 8)

Total body weights of the mice were assessed at the beginning and at the end of the study. As shown in Table 2, mice in each group gained weight regularly, and there were no differences in total body weight among the groups that received vehicle when compared with the ones treated with the drug. To investigate the effect of TPT on cognition, we used three different paradigms. In the Y-maze test, we observed that there was no difference in the general motor activities among the four groups of mice, as we found that the drug did not have any influence on the number of entries compared with controls (Fig. 1a). By contrast, we observed that compared with wild type mice (WT), 3xTg had a significant decrease in the percentage of alternations, which was significantly improved in the 3xTg treated with TPT (Fig. 1b). Interestingly, compared with WT controls, WT treated with TPT had also a significant increase in the percentage of alternation (Fig. 1b). In the fear conditioning test, we found that compared with WT, 3xTg mice had a significant decrease in freezing time for the cued, but not the contextual recall phase, and this was rescued in the 3xTg mice treated with the drug (Fig. 1c, d). During the 4 days training of the Morris water maze no differences were observed among the groups, and all mice were proficient swimmers (not shown). However, in the probe trial compared with WT the 3xTg mice had a reduction in the number of entries to the platform zone, and an increase of the latency to the first entry to the platform zone. By contrast, both deficits were significantly improved in the 3xTg treated with TPT (Fig. 1e-f). It is of interest to note that compared with WT controls, WT treated with the drug showed also more entries in the platform zone but the increase was not statistically significant (Fig. 1e).

**Table 2:** Total body weight (g) of the four group mice randomized to the study

Age	WT	WT/TPT	3xTg	3xTg/TPT
3 months	19.1 ± 0.44	17.7 ± 0.3	18.8 ± 0.37	18.2 ± 0.36
11 months	26.1 ± 1.08	25.7 ± 0.97	26.0 ± 0.8	26.0 ± 0.96
12 months	27.5 ± 0.96	27.3 ± 0.93	27.0 ± 0.8	26.9 ± 0.95

No significant differences were noted for any of the implemented behavioral tests when males and females were analyzed separately (Additional file 1: Figure S1 A, B).

### Pharmacological chaperone increases retromer complex levels

**Fig. 2:**
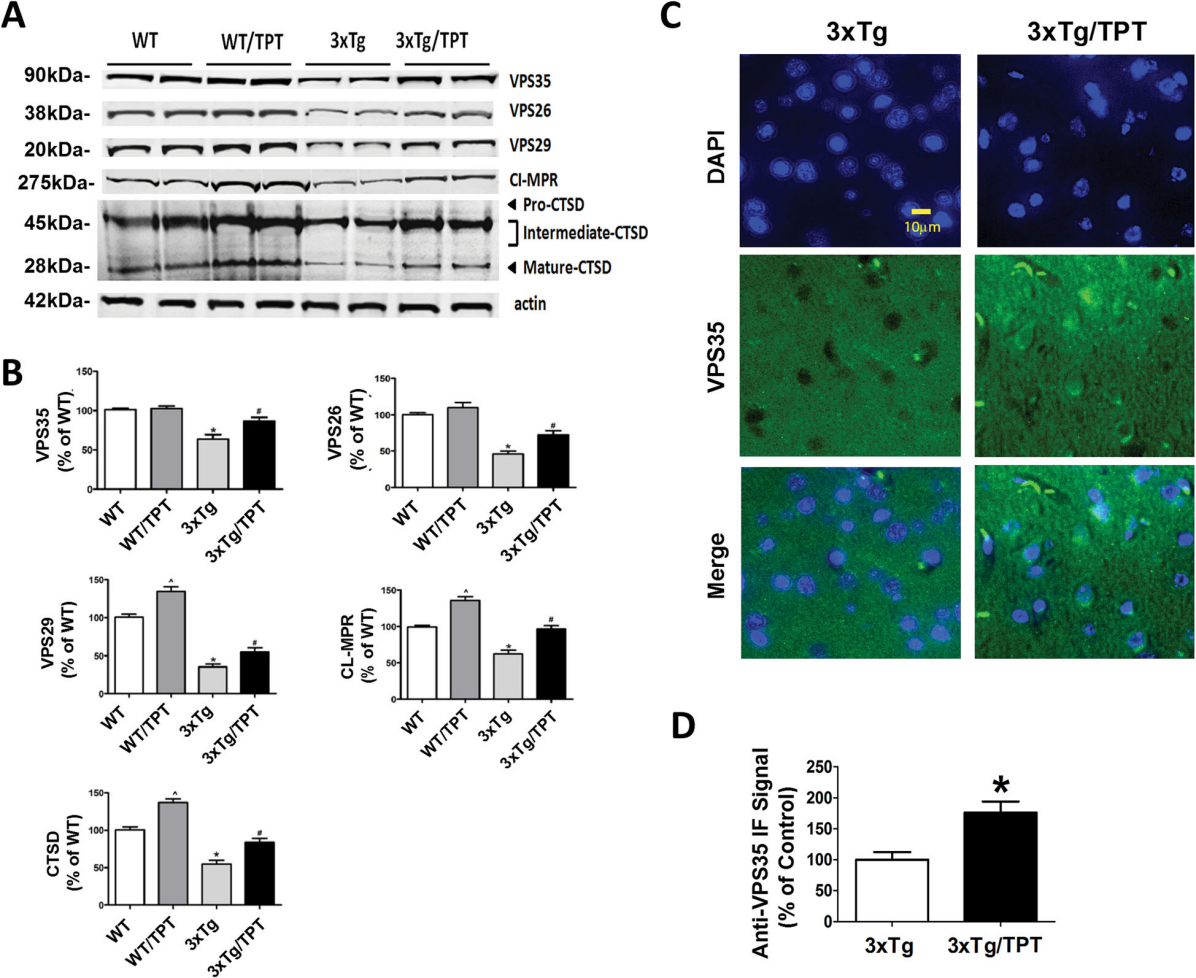
Pharmacological chaperone affects retromer complex levels in 3xTg mice. **a** Representative western blot analysis of VPS35, VPS26, VPS29, Cl-MPR and CTSD proteins in brain cortex homogenates from wild type (WT) and 3xTg mice treated with TPT or control (WT, 3xTg). **b** Densitometry of the immunoreactivities shown in the previous panel. Values represent mean ± standard error of the mean (*p < 0.05, WT Control vs WT/TPT, *n* = 3; #*p* < 0.05, WT Control vs 3xTg Control, n = 3; ^*p* < 0.05, 3xTg Control vs 3xTg/TPT, n = 3). **c**. Representative images of brain cortex sections of 3xTg receiving vehicle (3xTg) or TPT (3xTg/TPT) immunostained for VPS35 (scale bar 10 μm). **d** Quantification of the immune-fluorescent signal for VPS35 as observed in the previous panel. Values represent mean ± standard error of the mean (*p < 0.05 n = 3 per group)

Two weeks after the behavioral tests mice were euthanized, and brains assayed for the levels of the different proteins of the retromer complex system. As shown in Fig. 2, compared with WT, brain cortices of 3xTg had a significant decrease in the steady state level of the three components of the recognition core, VPS35, VPS26, and VPS29. However, these levels increased significantly in the brain of the treated mice (Fig. 2a, b). A similar effect was noted in the hippocampus (Additional file 2: Figure S2). Additionally, compared with WT brain cortices from 3xTg mice had lower level of a the cation-independent mannose 6-phosphate receptor (Cl-MPR) and cathepsin D (CTSD), both of which were significantly increased in drug treated 3xTg (Fig. 2a,b). Interestingly, compared with WT controls, TPT-treated WT mice had a significant increase in the steady state levels of VPS29, CI-MPR and CTDS (Fig. 2a, b). Immunofluorescence analysis confirmed the increase of VPS35 expression level in TPT-treated 3xTg mice (Fig. 2c, d).

### Pharmacological chaperone decreases Aβ burden

**Fig. 3:**
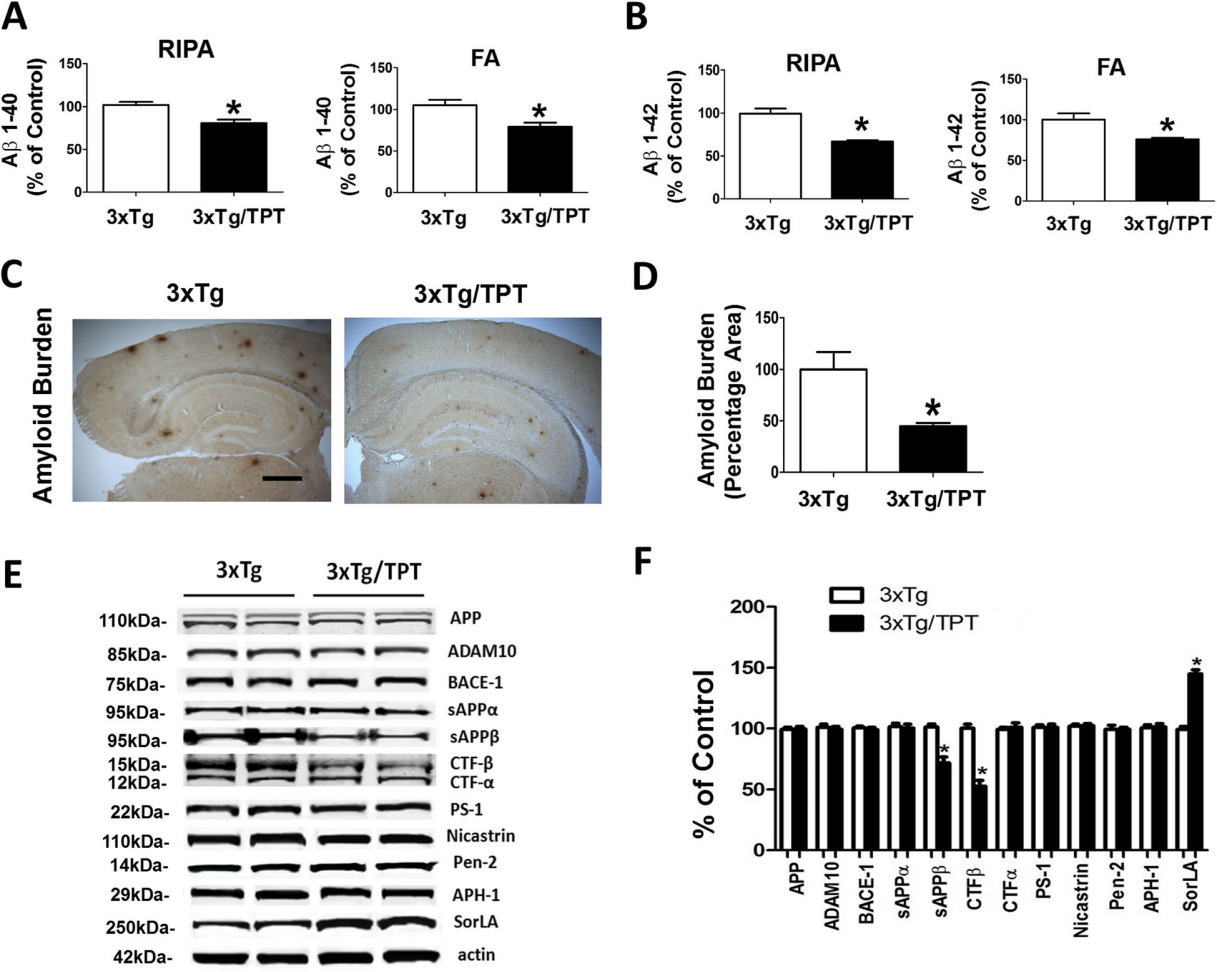
Pharmacological chaperone lowers Aβ levels and deposition in 3xTg mice. **a** Radioimmunoprecipitation assay (RIPA)-soluble and formic acid (FA)-extractable Aβ1–40 levels in cortex of 3xTg mice treated with TPT (3xTg/TPT) or controls (3xTg) were measured by sandwich enzyme-linked immunosorbent assay. Values represent mean ± standard error of the mean (*p < 0.05, *n* = 6). **b** RIPA-soluble and FA-extractable Aβ1–42 levels in cortex of 3xTg mice treated with TPT (3xTg/TPT) or controls (3xTg) were measured by sandwich enzyme-linked immunosorbent assay. Values represent mean ± standard error of the mean (*p < 0.05, n = 6). **c** Representative images of brain sections from 3xTg mice treated with TPT (3xTg/TPT) or control (3xTg) immuno-stained with 4G8 antibody to detect Aβ immunoreactivity (scale bar: 500 μm). **d** Quantification of the area occupied by Aβ immunoreactivity in brains of 3xTg mice treated with TPT or controls (3xTg). Values represent mean ± standard error of the mean (*p < 0.05, *n* = 4). **e** Representative Western blots of amyloid precursor protein (APP), sAPPα, sAPPβ, CTFs, BACE-1, ADAM10, APH-1, Nicastrin, Pen-2, PS1 and SorLA in cortex homogenates from 3xTg mice treated with TPT (3xTg/TPT) or controls (3xTg). **f** Densitometric analyses of the immunoreactivities to the antibodies shown in the previous panel. Values represent mean ± standard error of the mean (*p < 0.05, *n* = 3)

Compared with 3xTg controls, mice treated with TPT had a significant reduction of Aβ 1–40 and Aβ 1–42 levels in the RIPA-soluble and the formic acid-soluble fractions (Fig. 3a, b). Confirming these data, we found that the Aβ immuno-reactive areas in the brains of these animals were significantly decreased when compared with the control group (Fig. 3c). Because of these changes in Aβ peptides, next we investigated the metabolism of its precursor protein, APP, in order to identify potential mechanisms responsible for this effect. To this end, we assessed levels of APP, α-secretase (ADAM10), BACE-1 and the γ-secretase complex by western blot. Compared with controls, 3xTg mice treated with TPT had a significant reduction in the levels of sAPPβ and CTFβ (Fig. 3d, e). By contrast, we did not observe any differences between the two groups in the levels of APP, sAPPα , CTFα, ADAM10, BACE-1 and the γ-secretase complex (APH1, Pen2, PS1, Nicastrin) (Fig. 3d, e). Finally, compared with controls, 3xTg treated with TPT had a significant increase in the levels of SorLA (Fig. 3d, e).

### Pharmacological chaperone affects tau phosphorylation

**Fig. 4:**
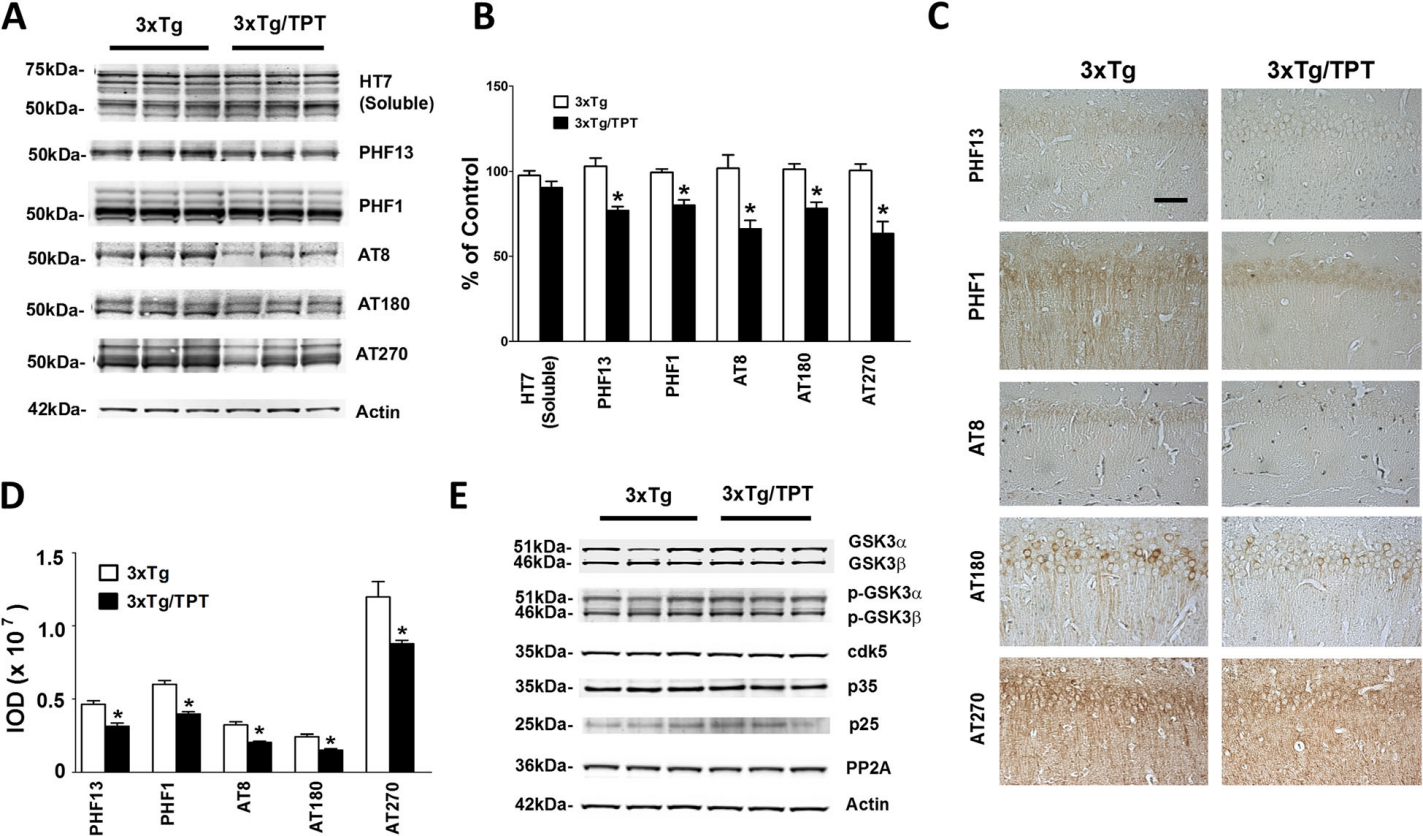
Pharmacological chaperone reduces tau phosphorylation in 3xTg mice. **a** Representative Western blots of total soluble tau (HT7), and phosphorylated tau at residues Ser396 (PHF13), Ser396/Ser404 (PHF1), Ser202/Thr205 (AT8), Thr231/Ser235 (AT180), and Thr181 (AT270) in brain cortex homogenates from 3xTg mice treated with TPT (3xTg/TPT) or control (3xTg). **b** Densitometric analyses of the immunoreactivities to the antibodies shown in the previous panel. Values represent mean ± standard error of the mean (*p < 0.05, n = 3). **c** Representative immuno-histochemical staining images for PHF13, PHF1, AT8, AT180 and AT270 positive areas in brain hippocampus sections of 3xTg mice treated with TPT or control (scale bar: 100 μm). **d** Quantification of the integrated optical density (IOD) for the immunoreactivity to the same antibody shown in panel C. Values represent mean ± standard error of the mean (*p < 0.05, n = 4). **e** Representative western blots of GSK3α, GSK3β, p-GSK-3α, p-GSK-3β, cdk5, p35, p25, and PP2A in brain cortex homogenates from 3xTg mice treated with TPT or control (Ctrl) (n = 4 per group)

Next, we evaluated the effect of TPT on tau levels and its phosphorylation at different epitopes. As shown in Fig. 4, no significant differences between the two groups were observed for the levels of total soluble tau (Fig. 4a, b). By contrast, we found that compared with controls, mice treated with TPT had a significant decrease in tau phosphorylated at epitopes: Ser396, Ser396/Ser404, Ser2020/Thr205, Thr231, and Thr181, as recognized by the PHF13, PHF1, AT8, AT180 and AT270 antibodies, respectively (Fig. 4a, b). Histochemical analyses confirmed the decrease in the immune-reactivity for the phosphorylated tau isoforms in brain sections of mice treated with TPT (Fig. 4c). In search for potential mechanism(s) underlying the TPT effect on tau phosphorylation, next we examined some of the kinases and phosphatases which are considered major regulators of these tau post-translational modifications. We found that compared with controls, brains of 3xTg mice treated with TPT did not have any changes in the levels of cdk5, p25, p35, total or phosphorylated GSK-3α, GSK-3β, and protein phosphatase 2A (PP2A) (Fig. 4d).

### Pharmacological chaperone influences synaptic integrity and neuroinflammation

**Fig. 5:**
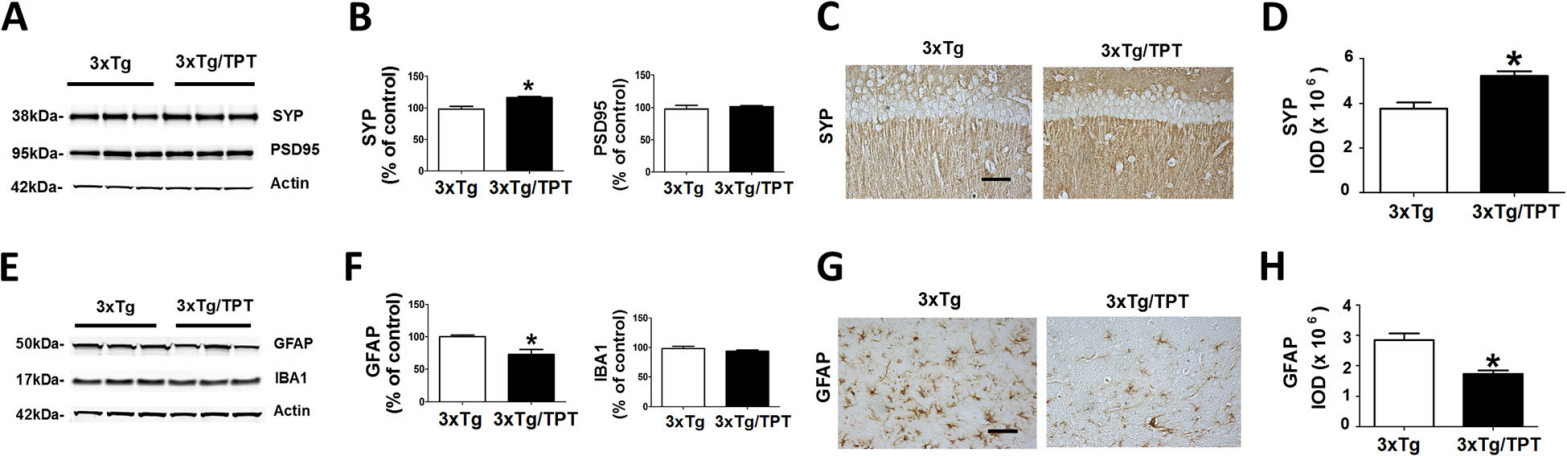
Pharmacological chaperone affects synaptic integrity and neuroinflammation in 3xTg mice. **a** Representative western blot analysis of synaptophysin (SYP), and post-synaptic density protein-95 (PSD-95) in brain cortex homogenates from 3xTg mice treated with TPT (3xTg/TPT) or control (3xTg). **b** Densitometric analyses of the immunoreactivities presented in the previous panel. Values represent mean ± standard error of the mean (*p < 0.05, n = 4). **c** Representative images of brain hippocampus sections from mice treated with TPT (3xTg/TPT) or control (3xTg) immuno-stained with SYP antibody (scale bar: 100 μm). **d** Quantification of the integrated optical density (IOD) for the immunoreactivity to the same antibody shown in panel **c**. Values represent mean ± standard error of the mean (*p < 0.05, n = 4). **e** Representative Western blot analyses of glial fibrillary acidic protein (GFAP) and IBA1 in brain cortex homogenates from 3xTg mice treated with TPT or control (3xTg). **f** Densitometric analyses of the immunoreactivities presented in the previous panel. Values represent mean ± standard error of the mean (*p < 0.05, n = 4). **g** Representative images of brain cortex sections from mice treated with TPT (3xTg/TPT) or control (3xTg) immuno-stained with GFAP antibody (scale bar: 100 μm). (H) Quantification of the integrated optical density for the immunoreactivity to the same antibody shown in panel **g**. Values represent mean ± standard error of the mean (*p < 0.05, n = 4 per group)

It is known that the memory impairments of the AD phenotype in this model are typically associated with altered markers of synaptic proteins. For this reason, we investigated whether the drug had any effect on them. Compared with controls, TPT-treated 3xTg mice had a significant elevation of synaptophysin (SYP), which was confirmed by immunohistochemistry (Fig. 5a-d). By contrast, no significant differences between the two groups were observed for post-synaptic density protein-95 (PSD-95) (Fig. 5a, b). Compared with controls, 3xTg treated with TPT had a statistically significant decrease in the levels of GFAP, a marker of astrocytes activation, which was also confirmed by immunohistochemistry (Fig. 5e-h). However, no significant differences were observed between the two groups when ionized calcium binding adaptor molecule 1 (IBA1), a marker of microglia activation, was assessed (Fig. 5e, f).

## Discussion

In this paper, we show that chronic administration of a pharmacological chaperone by upregulating VPS35 improves memory and synaptic integrity, lowers Aβ levels and deposition, decreases tau pathology and neuroinflammation in a transgenic mouse model of AD.

VPS35 is the major component of the cargo recognition module, a highly conserved trimer including also VPS26 and VPS29, which together with a dimer composed of different sorting nexin proteins (i.e., Snx1/Snx2) form a hetero-pentameric assembly known as the retromer complex system. The complex first described in the yeast is a ubiquitous and vital component of an intracellular trafficking and sorting mechanism for cargo proteins both in the recycling and retrieval pathways from the endosomes to different cell compartments [16]. Normal trafficking of different cargoes inside the cytoplasm is an essential activity for neuronal homeostasis, and evidence has accumulated that disruption of this system can alter APP metabolism and Aβ formation. Thus, as a transmembrane protein APP after being internalized is sorted in different cell compartments such as the endosomes from which reaches the trans-Golgi and then back the cell membrane [17]. Therefore retromer malfunction would interrupt the recycling process of APP resulting in its accumulation in the endosomes and a longer time for it to interact with BACE-1, which would result in increased Aβ production [18]. We and others have shown that genetic manipulation of VPS35 by interfering with this process modulates the phenotype of two AD mouse models, Tg2576 and 3xTg mice [7, 9]. Taken together these data suggest that VPS35 has a functional role in AD pathophysiology. However, in order for this new knowledge to have translational value it is imperative to demonstrate that VPS35 can be targeted pharmacologically.

Recently, a novel approach to assess the retromer involvement in neurodegeneration has been implemented by the use of pharmacological chaperones, molecules that stabilize folded and multimeric proteins by holding together their different parts against degradation and denaturation [19]. To this end, Mecozzi et al. identified two of these chaperones, which in neuronal cells stabilize the retromer recognition core module, and by doing so increase APP trafficking out of the endosomes resulting in less Aβ [10].

Taking advantage of this information, we designed our study in which we treated 3xTg mice, known to develop memory impairments, Aβ plaques and tau tangles, with the chaperone TPT and then assessed its effects on the phenotype. Chronic administration of the drug had no effect on the general health of the mice, and no differences in total body weight were observed when untreated mice were compared with the treated ones. To assess the effect of TPT on memory, mice were tested in several behavioral paradigms. Initially, we implemented the Y-maze paradigm, which assesses working memory in rodents. In this test, as predicted the untreated 3xTg mice performed worse than the untreated WT mice since they had a lower percentage of alternations. However, this deficit was significantly reduced in the 3xTg mice receiving the pharmacological chaperone. Interestingly, we observed that also the WT mice treated with TPT had a significant improvement in the percentage of alternations, suggesting that the pharmacological effect is not transgene dependent. By contrast, no effect of the drug on any of the groups was observed when the entries in the arms of the maze were assessed indicating that the drug did not influence their motor activity [20]. In the fear conditioning test, a measure of associative learning and memory, while 3xTg controls had significantly less freezing time than the WT, the 3xTg mice treated with TPT had a significant improvement in the cued phase of the test, which reflects a non-hippocampal component in this paradigm [21]. Finally, mice were assessed in the Morris water maze which measures spatial learning and memory. In this test, while we did not observe any differences among the groups during the four-days training sessions, in the probe trial we found that compared with WT controls, 3xTg had a significant reduction in the number of entries in the platform zone, and an increase in the latency to enter the platform zone. By contrast, TPT-treated 3xTg had a significant amelioration for both parameters. Interestingly, drug treated WT mice had also an improvement in the number of entries, further supporting that the effect of the compound is not transgene dependent.

Confirming compliance with the drug regimen, we observed that TPT-treated mice had a significant increase in the levels of VPS35, which associated with a similar increment in the other two recognition core proteins, VPS26 and VPS29. This effect does not come as a surprise, since previous works have shown that manipulation of one of the three components of this hetero-trimer module results into secondary changes of the others two, suggesting that the interaction among the single unit is important for complex function and stability [22–24]. Interestingly, we also observed that other components of the complex system were affected by the stabilization of VPS35, namely CI-MPR and CTSD. This fact supports the therapeutic efficacy of the drug we used in our study, since CI-MPR is known as the receptor for the transport of mature CTSD from the Golgi to the endosomes before reaching the final destination, the lysosomes [25, 26].

The up-regulation of VPS35 and restoration of the retromer complex system function resulted in significant Aβ reduction in the brain parenchyma of the treated mice. Having observed this change, next we investigated whether the drug had an effect on APP processing. While no significant changes were found in the proteases involved in its cleavage, TPT-treated mice had a significant reduction in the BACE-1-derived products sAPPβ and CTF-β, suggesting that the effect on Aβ levels were secondary to a decreased proteolytic action of BACE-1 on APP. We also observed that treated mice with TPT had an increase of SorLA, an important protein implicated in APP transport from the endosome to the Golgi [27]. While we do not know the exact mechanism underlying this biological effect, we speculate that it is most likely secondary to the increased levels of VPS35. Thus, previous work has clearly shown that the retromer can interact and have a direct impact on SorLA expression and function at the endosomal level both in neurons and microglia cells [28].

Since the mouse model implemented develops progressive accumulation of phosphorylated tau, next we assessed the effect of TPT on this aspect of the phenotype. Compared with the untreated group, the one receiving the drug had a significant reduction in phosphorylated tau at different epitopes. In search for possible mechanisms responsible for this effect, we looked at some of the kinases which have been implicated in this post-translational modification. No differences were found between the two groups when both the cdk5, GSK3 pathways were investigated, and a similar result was also observed when a major phosphatase, PPA2, was assessed. These findings support the hypothesis that the TPT-dependent changes in tau phosphorylation were secondary to a better turn-over and degradation of this protein resulting from the restoration of the retromer complex trafficking and sorting functions. Thus, having observed a significant increase in the CTSD and its receptor CI-MPR we speculate that the higher availability of this protease, previously involved in tau degradation [29], could be responsible for the lower levels of pathological tau in the treated mice [30].

Having observed an amelioration in the cognitive performance secondary to the implemented therapy, next we assessed synapse integrity. Compared with untreated mice, we found that the treated ones had a significant elevation in the levels of synaptophysin, a pre-synaptic protein marker, underscoring the biochemical substrate for the observed behavioral improvements [31]. Finally, we investigated whether our therapeutic regimen had an effect on neuroinflammation, another important aspect of the AD phenotype. To this end, we assayed the levels of two markers of inflammation: GFAP for astrocytes, and IBA1 for microglia. Biochemical and immunohistochemical analyses showed that compared with the controls, TPT-treated 3xTg mice had lower levels of GFAP, suggesting a decrease in astrocytes activation [32].

## Conclusions

Our investigation demonstrates that chronic administration of a pharmacological chaperone positively influences all the major phenotypic aspects of a mouse model of AD: memory deficits, synapse, Aβ and tau pathology, and neuroinflammation. Collectively, our findings further support the active role and direct impact that the retromer complex has on AD pathophysiology by modulating both APP and tau metabolism. Importantly, our study represents a proof of principle for the druggability of VPS35 and supports the exciting possibility that, since this component of the recognition core can be targeted in vivo by pharmacological chaperone, it represents a novel and viable therapeutic approach against AD.

## Supplementary information

**Additional file 1: Figure S1. A.** Effect of TPT on behavioral tests in male mice. (A) Number of total arm entries for wild-type mice (WT) and 3xTg mice (3xTg) treated with TPT or control (Ctrl). (B) Percentage of alternations for WT and 3xTg mice treated with TPT or control (Ctrl). (C) Contexual fear memory response in WT and 3xTg mice treated with TPT or control (Ctrl). (D) Cued fear memory response in WT and 3xTg mice treated with TPT or control (Ctrl). (E) Morris water maze, probe trial for the same four groups of mice, number of entries to the platform area; (F) Morris water maze, probe trial for the same four groups of mice, latency to first entry to the platform area. Values represent mean ± standard error of the mean. (#*p* < 0.05, WT Control vs 3xTg Control; ^*p* < 0.05, WT/TPT vs 3xTg/TPT). (WT Control: *n* = 4; WT/TPT: *n* = 5; 3xTg Control, n = 4; 3xTg/TPT, n = 4). **B.** Effect of TPT on behavioral tests in female mice. (A) Number of total arm entries for wild-type mice (WT) and 3xTg mice (3xTg) treated with TPT or control (Ctrl). (B) Percentage of alternations for WT and 3xTg mice treated with TPT or control (Ctrl). (C) Contexual fear memory response in WT and 3xTg mice treated with TPT or control (Ctrl). (D) Cued fear memory response in WT and 3xTg mice treated with TPT or control (Ctrl). (E) Morris water maze, probe trial for the same four groups of mice, number of entries to the platform area; (F) Morris water maze, probe trial for the same four groups of mice, latency to first entry to the platform area. Values represent mean ± standard error of the mean. (#*p* < 0.05, WT Control vs 3xTg Control; ^*p* < 0.05, WT/TPT vs 3xTg/TPT). (WT Control: n = 4; WT/TPT: n = 5; 3xTg Control, n = 4; 3xTg/TPT, *n* = 4).

**Additional file 2: Figure S2.** Pharmacological chaperone affects retromer complex levels in hippocampi of 3xTg mice. (A) Representative western blot analysis of VPS35, VPS26b and VPS29 proteins in hippocampus homogenates from 3xTg mice treated with TPT or control (Ctrl). (B) Densitometry of the immunoreactivity shown in the previous panel. Values represent mean ± standard error of the mean. (*p* < 0.05; *n* = 4 animals per group).

## Data Availability

Raw data is available from the corresponding authors upon reasonable request.

## Abbreviations

AD: Alzheimer’s disease
APP: Amyloid beta precursor protein
Aβ: Amyloid β
BACE-1: Beta secretase 1
CI-MPR: Cation independent mannose 6-phosphate receptor
CTF-β: C-terminal fragment β
CTSD: Cathepsin D
GFAP: Glial fibrillary acidic protein
IBA1: Ionized calcium binding adaptor molecule 1
RIPA: Radio-immuno-precipitation assay
sAPPα: Secreted APP alpha
sAPPβ: Secreted APP beta
Tg: Transgenic
VPS26: Vacuolar sorting protein 26
VPS29: Vacuolar sorting protein 29
VPS35: Vacuolar sorting protein 35
WT: Wild type
