# Correction to: A pharmacological chaperone improves memory by reducing Aβ and tau neuropathology in a mouse model with plaques and tangles

**DOI:** 10.1186/s13024-021-00461-4

**Published:** 2021-06-28

**Authors:** Jian-Guo Li, Jin Chiu, Mercy Ramanjulu, Benjamin E. Blass, Domenico Praticò

**Affiliations:** 1 Alzheimer’s Center at Temple, Lewis Katz School of Medicine, Temple University, 3500 North Broad Street, MERB, suite 1160, Philadelphia, PA 19140 USA; 2 Moulder Center for Drug Discovery Research, School of Pharmacy, Temple University, Philadelphia, PA 19140 USA

**Correction to: Mol Neurodegeneration 15, 1 (2020)**

**https://doi.org/10.1186/s13024-019-0350-4**

After publication of the original article [1], we have been alerted for errors in 2 figure panels. Specifically, in the original Fig. 3E the lanes probed for ADAM-10, Nicastrin, APH-1 and actin were run on different gels. During the first revision of the paper, we actually realized this error and re-run the gels for these proteins so that we could have corrected the figure. The results confirmed the findings, but unfortunately, we did not replace the original representative images in the final version of this panel. Here we want to apologize for the errors appeared in this publication. Despite the errors in the previous data presentation, the original conclusion is sustained as the experiments have been repeated in the lab. The corrected panels have now been replaced in the amended figure below.

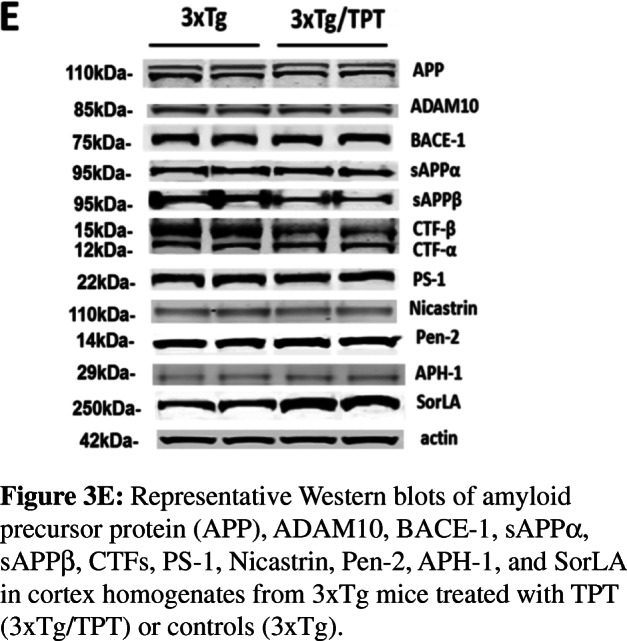



In the original Fig. 4E, the lane probed for actin was accidentally placed also in the lane probed for cdk5. This error was not captured in the final version due to our negligence, and we are sorry for this careless mistake. The correct full-blot images for this set of Western blots were included in a supplementary file during peer-review. This file wasn’t included in the paper during the production for the online publication. The correct panel has been inserted in the amended figure below. Again, the conclusion is not impacted by this replacement.

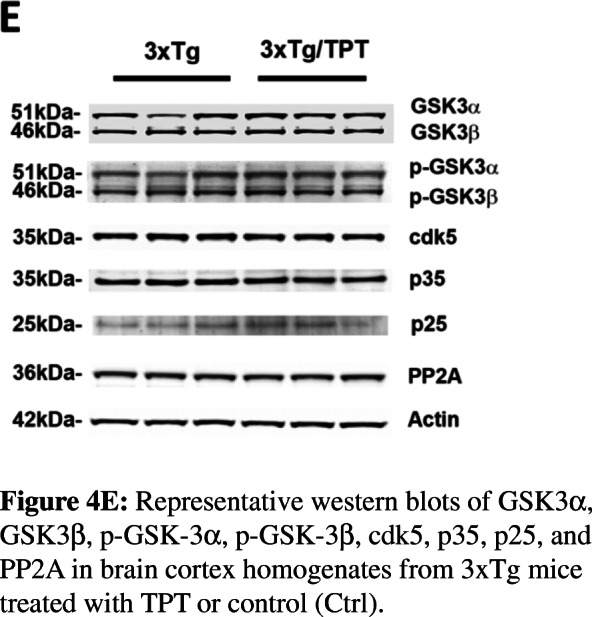



The authors sincerely apologize for these mistakes and any inconvenience that these errors may have created to the readers.
