# Supplementary figures and images for: A pharmacological chaperone improves memory by reducing Aβ and tau neuropathology in a mouse model with plaques and tangles
Source: Mol Neurodegener. 2020 Jan 22;15:1. doi: 10.1186/s13024-019-0350-4 (PMC6975032; doi:10.1186/s13024-019-0350-4)

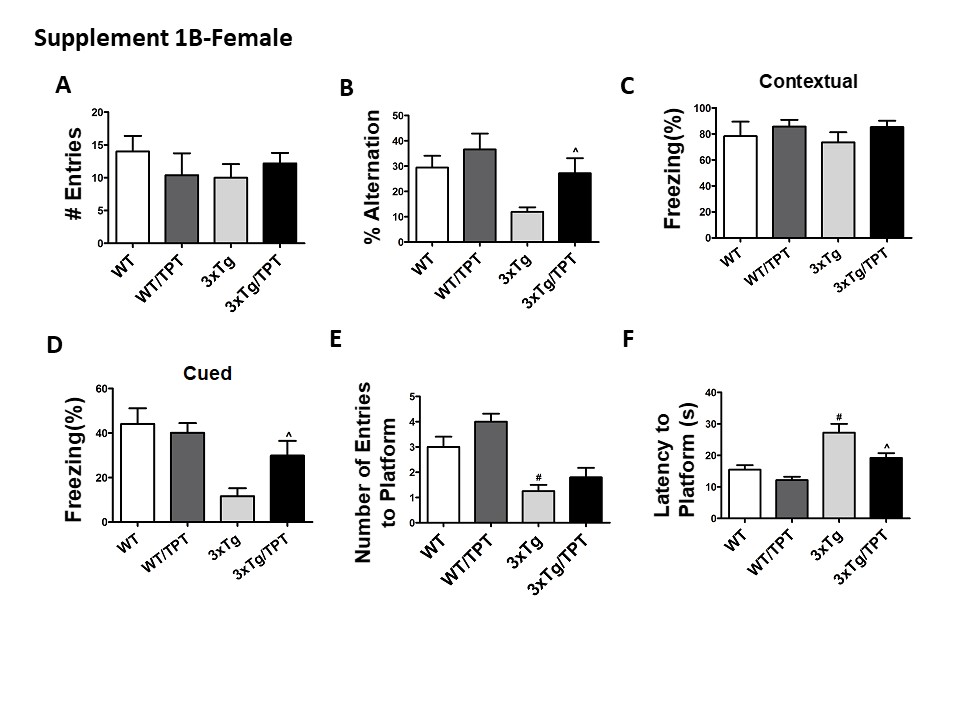

Supplement: Supplementary file 1 — Additional file 1: Figure S1. A. Effect of TPT on behavioral tests in male mice. (A) Number of total arm entries for wild-type mice (WT) and 3xTg mice (3xTg) treated with TPT or control (Ctrl). (B) Percentage of alternations for WT and 3xTg mice treated with TPT or control (Ctrl). (C) Contexual fear memory response in WT and 3xTg mice treated with TPT or control (Ctrl). (D) Cued fear memory response in WT and 3xTg mice treated with TPT or control (Ctrl). (E) Morris water maze, probe trial for the same four groups of mice, number of entries to the platform area; (F) Morris water maze, probe trial for the same four groups of mice, latency to first entry to the platform area. Values represent mean ± standard error of the mean. (#p < 0.05, WT Control vs 3xTg Control; ^p < 0.05, WT/TPT vs 3xTg/TPT). (WT Control: n = 4; WT/TPT: n = 5; 3xTg Control, n = 4; 3xTg/TPT, n = 4). B. Effect of TPT on behavioral tests in female mice. (A) Number of total arm entries for wild-type mice (WT) and 3xTg mice (3xTg) treated with TPT or control (Ctrl). (B) Percentage of alternations for WT and 3xTg mice treated with TPT or control (Ctrl). (C) Contexual fear memory response in WT and 3xTg mice treated with TPT or control (Ctrl). (D) Cued fear memory response in WT and 3xTg mice treated with TPT or control (Ctrl). (E) Morris water maze, probe trial for the same four groups of mice, number of entries to the platform area; (F) Morris water maze, probe trial for the same four groups of mice, latency to first entry to the platform area. Values represent mean ± standard error of the mean. (#p < 0.05, WT Control vs 3xTg Control; ^p < 0.05, WT/TPT vs 3xTg/TPT). (WT Control: n = 4; WT/TPT: n = 5; 3xTg Control, n = 4; 3xTg/TPT, n = 4). [file 13024_2019_350_MOESM1_ESM.zip › Jian Guo 3xtG -TPT-paper supple Fig 1B.jpg]

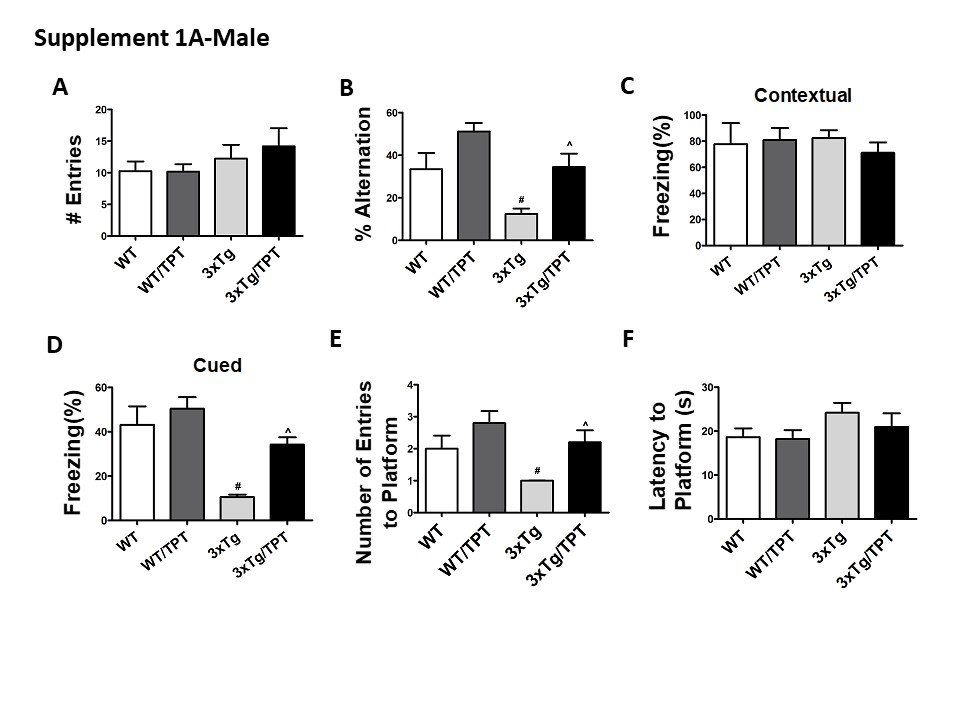

Supplement: Supplementary file 1 — Additional file 1: Figure S1. A. Effect of TPT on behavioral tests in male mice. (A) Number of total arm entries for wild-type mice (WT) and 3xTg mice (3xTg) treated with TPT or control (Ctrl). (B) Percentage of alternations for WT and 3xTg mice treated with TPT or control (Ctrl). (C) Contexual fear memory response in WT and 3xTg mice treated with TPT or control (Ctrl). (D) Cued fear memory response in WT and 3xTg mice treated with TPT or control (Ctrl). (E) Morris water maze, probe trial for the same four groups of mice, number of entries to the platform area; (F) Morris water maze, probe trial for the same four groups of mice, latency to first entry to the platform area. Values represent mean ± standard error of the mean. (#p < 0.05, WT Control vs 3xTg Control; ^p < 0.05, WT/TPT vs 3xTg/TPT). (WT Control: n = 4; WT/TPT: n = 5; 3xTg Control, n = 4; 3xTg/TPT, n = 4). B. Effect of TPT on behavioral tests in female mice. (A) Number of total arm entries for wild-type mice (WT) and 3xTg mice (3xTg) treated with TPT or control (Ctrl). (B) Percentage of alternations for WT and 3xTg mice treated with TPT or control (Ctrl). (C) Contexual fear memory response in WT and 3xTg mice treated with TPT or control (Ctrl). (D) Cued fear memory response in WT and 3xTg mice treated with TPT or control (Ctrl). (E) Morris water maze, probe trial for the same four groups of mice, number of entries to the platform area; (F) Morris water maze, probe trial for the same four groups of mice, latency to first entry to the platform area. Values represent mean ± standard error of the mean. (#p < 0.05, WT Control vs 3xTg Control; ^p < 0.05, WT/TPT vs 3xTg/TPT). (WT Control: n = 4; WT/TPT: n = 5; 3xTg Control, n = 4; 3xTg/TPT, n = 4). [file 13024_2019_350_MOESM1_ESM.zip › Jian Guo 3xtG -TPT-paper supple Fig 1A.jpg]

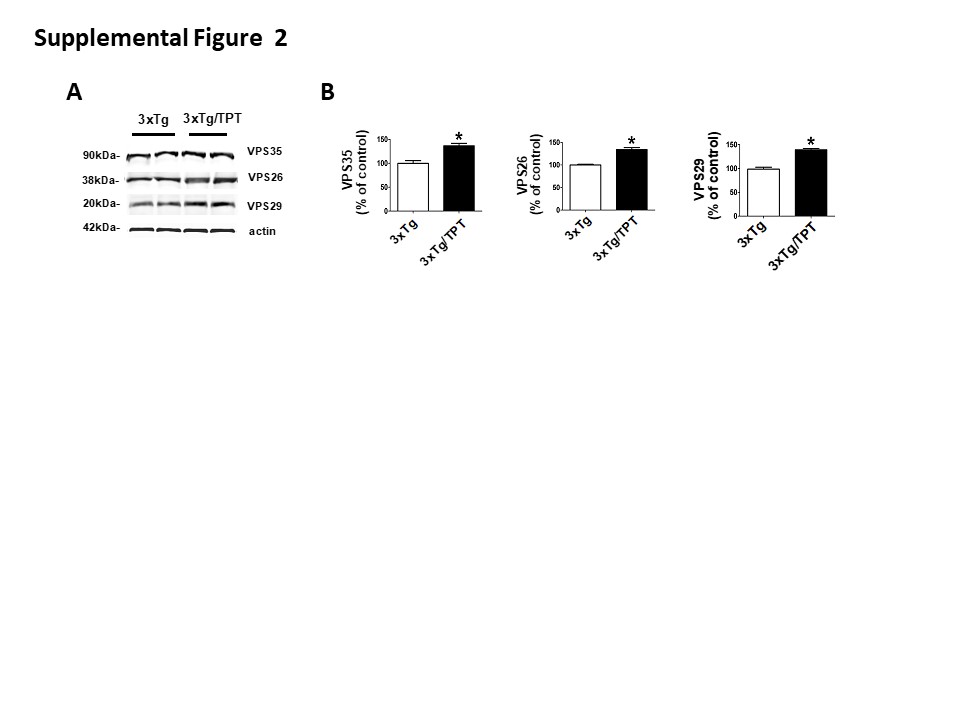

Supplement: Supplementary file 2 — Additional file 2: Figure S2. Pharmacological chaperone affects retromer complex levels in hippocampi of 3xTg mice. (A) Representative western blot analysis of VPS35, VPS26b and VPS29 proteins in hippocampus homogenates from 3xTg mice treated with TPT or control (Ctrl). (B) Densitometry of the immunoreactivity shown in the previous panel. Values represent mean ± standard error of the mean. (p < 0.05; n = 4 animals per group). [file 13024_2019_350_MOESM2_ESM.jpg]
